# Parsing the Role of PPARs in Macrophage Processes

**DOI:** 10.3389/fimmu.2021.783780

**Published:** 2021-12-22

**Authors:** Daniel Toobian, Pradipta Ghosh, Gajanan D. Katkar

**Affiliations:** ^1^ Department of Cellular and Molecular Medicine, University of California San Diego, San Diego, CA, United States; ^2^ Rebecca and John Moore Comprehensive Cancer Center, University of California San Diego, San Diego, CA, United States; ^3^ Department of Medicine, University of California San Diego, San Diego, CA, United States; ^4^ Veterans Affairs Medical Center, La Jolla, CA, United States

**Keywords:** macrophage, PPAR, PPAR agonist, inflammatory disease, PPAR a, PPAR γ

## Abstract

Cells are richly equipped with nuclear receptors, which act as ligand-regulated transcription factors. Peroxisome proliferator activated receptors (PPARs), members of the nuclear receptor family, have been extensively studied for their roles in development, differentiation, and homeostatic processes. In the recent past, there has been substantial interest in understanding and defining the functions of PPARs and their agonists in regulating innate and adaptive immune responses as well as their pharmacologic potential in combating acute and chronic inflammatory disease. In this review, we focus on emerging evidence of the potential roles of the PPAR subtypes in macrophage biology. We also discuss the roles of dual and pan PPAR agonists as modulators of immune cell function, microbial infection, and inflammatory diseases.

## Introduction

**Figure 1:**
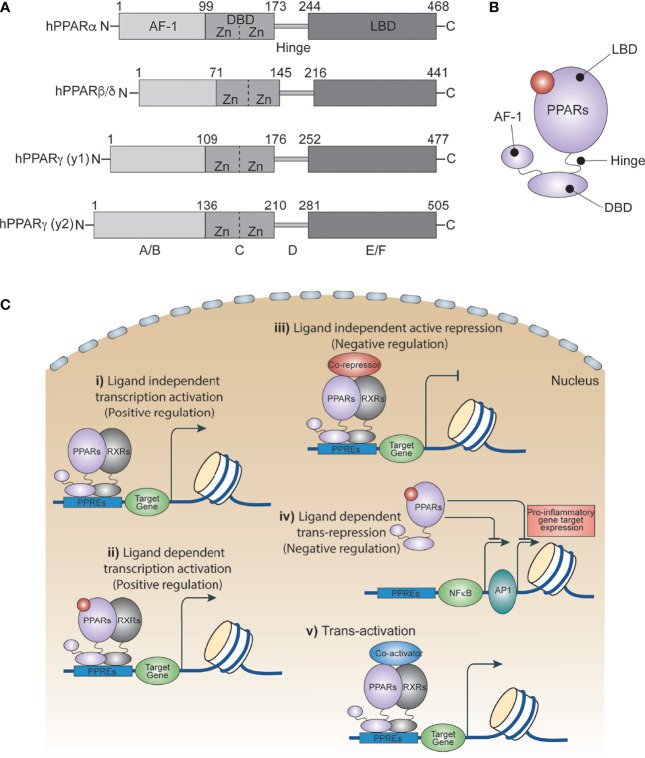
PPAR isoforms and their transcriptional regulatory function. **(A)** A schematic of the domain architect of PPAR isoforms PPARα, PPARβ/δ and PPARγ. **(B)** Cartoon showing ligand binding site in PPARs. **(C)** Ligand independent and dependent transcriptional regulatory mechanism of PPARs.

Peroxisome proliferator activated receptors (PPARs) are ligand-dependent transcription factors that are structurally conserved members of the nuclear receptor superfamily (1). PPARs influence a variety of cell signals including cellular differentiation and development (2–4), lipid metabolism (5), the insulin signaling network (6), homeostasis (7) and tumorigenesis (2, 3, 8). In 1960, scientists showed an increased number of peroxisomes in the livers of rats treated with hypolipidemic drugs. A decade later, this increase was attributed to certain members of the nuclear receptor family. In 1990, Issemann and Green cloned these receptors for the first time and demonstrated that hepatocarcinogens promote the proliferation of peroxisomes in rodents through these receptors, and thus named them Peroxisome Proliferator Activated Receptors (PPARs) (9). Three PPAR isoforms have been identified thus far: PPARα, PPARβ/δ and PPARγ. They each have distinct patterns of function and tissue distribution, and are expressed in various cell types including immune cells (6, 10, 11), epithelial cells (12) and endothelial cells (13, 14). All PPARs utilize a common domain organization (**Figure 1A**) with a slightly variable amino-terminal that contributes to transcriptional activation function, and a central highly conserved DNA binding domain that contains a zinc motif (15). A ligand-binding domain at the carboxy-terminal end confers their ligand-binding property, regulates ligand-dependent transcriptional activation and repression functions, and contributes to receptor homo- or heterodimerization (**Figures 1B** **, C**) (16, 17).

PPARγ is the most extensively characterized and researched member of the PPAR subfamily and consists of two isoforms, PPARγ1 and PPARγ2, that are expressed mainly in adipose tissue. They mediate adipocyte differentiation by regulating the expression of genes that are involved in lipid metabolism and insulin signaling (18). PPARα is the second most studied PPAR, expressed mainly in liver and immune cells which regulate lipid catabolism, especially during fasting conditions (19). The third is PPARδ, (aka PPARβ) which is highly active in skeletal muscle where it is also involved in regulating fatty acid catabolism (20, 21). PPARδ activation also increases insulin sensitivity, improves lipid homeostasis, and prevents weight gain. Though PPARs are extensively studied, their role in molecular and cellular signaling in immune cells has limited understanding.

PPARs also regulate the functions of the innate immune system such as macrophage function and differentiation (5, 17, 22, 23). Thus, there has been substantial interest in understanding and defining the functions of PPARs and their agonists in regulating gene expression in macrophage biology and how that relates to acute and chronic inflammatory diseases (22). Few studies attempted to discuss the role of PPARs in macrophage function, and the discussion is generally limited to PPARγ (6, 10, 22, 24). Since all three isoforms of PPARs regulate each other’s expression through feedback loops, it is worthwhile to understand their role together. In this review, we begin with a brief introduction of PPAR signaling and mechanism, and then highlight recent developments that provide insight into how isoforms of PPAR and their agonists can regulate several steps involved in the initiation, proliferation, and resolution of inflammatory responses in macrophages, especially in the context of microbial infection and inflammatory diseases.

## Transcriptional Mechanism of PPARs

**Figure 2:**
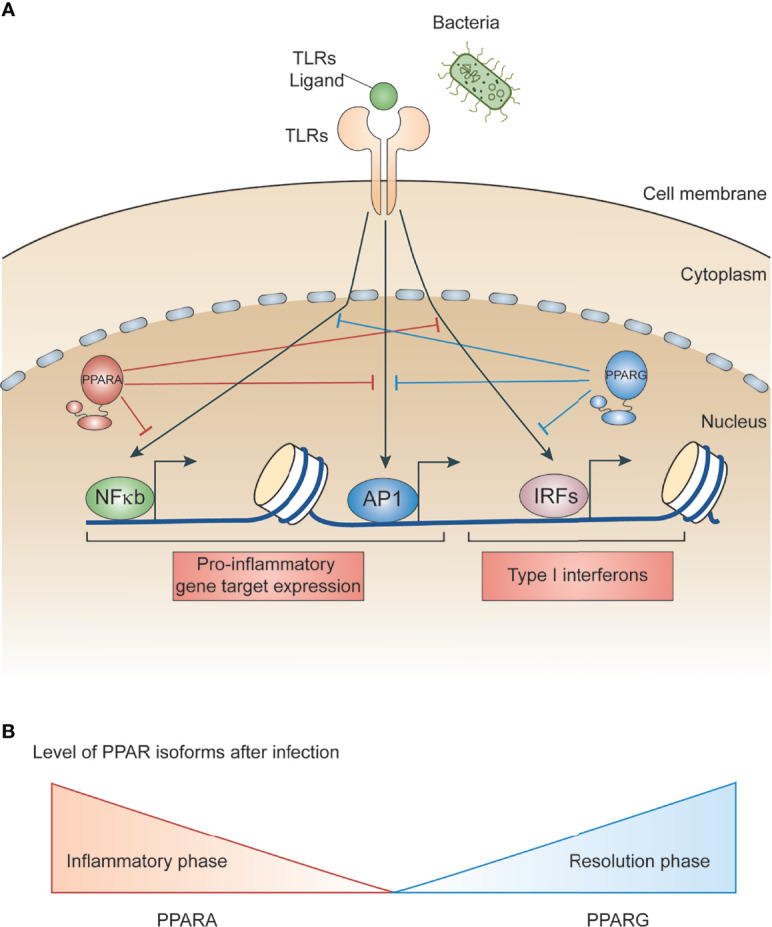
Role of PPARs in bacteria-induced inflammatory signaling. **(A)** Schematic illustrating LPS/bacteria induced inflammatory signal promoting transcription factors. Whereas PPARs interacts with and modulates transcription factors involved in microbe induced inflammation. **(B)** Schematic depicting gene expression of PPARs during infection. PPARA level is higher during inflammatory phase whereas PPARG is higher during resolution phase.

PPARs regulate several metabolic and inflammatory signaling pathways during infection through both positive and negative regulation of gene transcription (22, 25). The positive regulation comes from direct binding of PPARs to peroxisome proliferator hormone response elements (PPREs) present in the vicinity of target genes. PPARs predominantly bind as heterodimers with retinoid X receptors (RXRs), either in presence or absence of ligands (**Figure 1C**), to stimulate transcription activity (23, 25) For example, PPARα increases expression of carnitine palmitoyl transferase (CPT)-I, an enzyme located in the mitochondrial outer membrane controlling fatty acid β-oxidation (23, 25),. Additionally, PPARs negatively regulate target genes by constitutively binding, along with nuclear co-repressors, to the PPREs of target genes which often function as transcriptional repressors in absence of ligands (**Figure 1C**). For example, NCoR and SMRT decrease transcriptional activity of PPARγ thus preventing iNOS induction by LPS. Also, PPARs bind directly to transcriptional factors involved in inflammation including NF-κB and AP1, inhibiting their transcriptional activity. This phenomenon is termed as ‘trans-repression’ (**Figures 1C**, **2A, B**) (23, 25).

Trans-activation is mainly regulated by recruiting co-activators (**Figure 1C**), which enhance activation of PPAR-regulated genes. For example, PPAR coactivator-1*α* (PGC-1*α*) is a co-activator of both PPARα and PPARγ. Repression, trans-repression, and trans-activation mechanisms of transcriptional control of PPARs has been extensively studied and reviewed elsewhere. In the following section, we discuss recent progress in understanding how PPARs and their agonists regulate the metabolic and inflammatory signaling of macrophages in response to infection and inflammatory diseases.

## Inflammation and Infection

Inflammation is a host response that targets invading infectious agents and tissue injury through recruitment of immune cells and repair machinery. Macrophages detect pathogen associated molecular patterns (PAMPs) present on microbes using pattern recognition receptors (PRR) (26–28). For example, toll-like receptor 4 (TLR4) is a PRR that recognizes lipopolysaccharides (LPS) present on gram-negative bacteria cell walls (28). In addition to recognizing diverse microbial components, many toll-like receptors also detect endogenous danger signals associated with tissue injuries and inflammatory diseases. Upon microbial invasion or purified ligand stimulation, TLRs promote rapid activation of major signal dependent transcription factors: nuclear factor-κB (NF-κB), activator protein 1 (AP1), and interferon regulatory factors (IRFs) (10, 28) (**Figure 2A**). These transcription factors work together to rapidly induce genes that trigger the initial inflammatory response, promote antimicrobial activity, and activate development of acquired immunity. It is important for macrophages to sustain sufficient inflammation to kill invading microbes. This sustained inflammation is maintained by several cytokines upregulated during initial stimulation which promote a forward transcriptional loop due to the autocrine and paracrine effects of cytokines (26, 29, 30). However, sustained inflammation can lead to collateral tissue damage (30). Therefore, negative feedback loops are essential to limit the extent of inflammation and promote resolution.

## The Lineage-Determining Role of PPARs in Tissue-Resident Macrophage Populations

**Figure 3:**
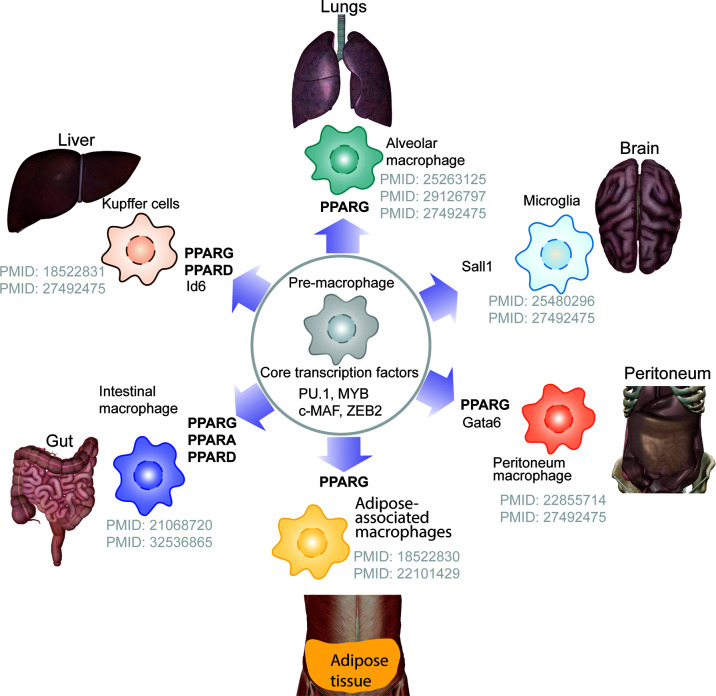
PPARs and tissue-resident macrophage. The schematics depicts the lineage determining role of PPARs in tissue resident macrophage, showing PPARG but not PPARA is major transcription that contributes towards defining the tissue-resident macrophage.

Tissue-resident macrophages support embryonic development and tissue homeostasis. During early embryonic stage pre-macrophage are colonize entire embryo and rapidly diversify transcription programme depending on tissue specific transcription factor need. PPARs, most notably PPARγ, play a role in defining the lineage of tissue-resident macrophages, whereas other PPAR isoforms contribute lesser. PPARγ is required for the transcriptional modulation in regulating differentiation of pre-macrophages to alveolar macrophages (31, 32), Kupffer cells (33), adipose-associated macrophages, and intestinal macrophages (**Figure 3**). It has been demonstrated that granulocyte-macrophage colony-stimulating factor (GM-CSF) promotes the expression of PPARγ, one of the major transcription factors regulating differentiation of pre-macrophage to alveolar macrophages (31, 32). Although, molecular mechanism underlying role PPARs in lineage determination of tissue macrophage deserves to be explored, several studies, in the past have reported the role of PPARs in macrophage polarization.

## Macrophage Polarization and PPARs

Macrophages are phagocytic innate immune cells, whose functions include scavenging microbes and apoptotic and necrotic cells, as well as playing a role in lipid homeostasis and tissue remodeling. Macrophages possess functional heterogeneity, in that they uptake different functions depending on signaling factors and metabolic changes (26–28, 30). For example, upon sensing invading pathogens and Th-1 cytokines such as IFNγ, macrophages assume immune reactive form (pro-inflammatory/classical activation state, M1) to phagocytose pathogens. In contrast, after encountering Th-2 cytokines such as IL4 and IL13, macrophages assume immune tolerant form (anti-inflammatory/alternative activation state, M2) to help with tissue repair and angiogenesis in injured tissues (27, 30). Impaired functions of both immune reactive and immune tolerant states of macrophages could lead to host tissue damage and development of chronic disease (10, 30, 34–36).

PPARγ activation suppresses the immunoreactive state of macrophage as reported by suppression of immune reactive cytokine markers such as NOS2, TNFα, IL6, IL1β and MCP1 in murine macrophages (37), whereas its activation promotes immunotolerant state markers such as CD36, IL13, Arg1, Ym1, Fizz1, CD206, IL4, and IL10 in murine macrophages (22, 38–41). PPARγ deficient mouse macrophages also showed an increase in Th1 cytokines such as TNF-α, IL1-β, IL-6, IL-12 and a reduction of Th2 cytokine IL10 when induced with LPS (42). PPARγ also inhibits the expression of HIF1a, which plays key role in inducing the immune reactive phenotype, and promotes Arginase 1 expression, which is a hallmark marker of the immune tolerant macrophage, in mice (43). The evidence makes it clear that PPARγ is in charge of, or at least promotes, the immune tolerant state of macrophages. This is further evidenced by how PPARγ responds to infection *in vivo*.

As stated, the function of immune tolerant macrophages includes post-infection repair, which includes the cleanup of debris (44). PPARγ agonists have been shown to increase Fcγ receptor-mediated opsonized phagocytosis in murine alveolar macrophages (45, 46) demonstrating a possible pathway in which PPARγ controls the cleanup process. In terms of repair, one study showed that PPARγ deficient mice had an increase in pulmonary collagen deposition following influenza infection (47), demonstrating PPARγ’s role is proper tissue repair post-infection. Again, PPARγ shows itself to be a good promoter of the immune tolerant macrophage phenotype.

PPARγ can also affect macrophage polarization in a ligand-independent manner through trans-activation. For example, after alternative activation through exposure to IL4, macrophages displayed a remodeled and more accessible chromatin profile, an upregulation of PPARγ, and no changes in RXR levels. Upon subsequent stimulation with IL4, PPARγ bound to DNA independently of ligands through the recruitment of P300 and RAD21, leading to further anti-inflammatory activity (48). Additionally, PPARγ transcriptional activity has been induced in a ligand-independent manner by insulin and C-peptide. Neither insulin nor C-peptide affected PPARγ transcription levels. Also, the addition of PPARγ antagonist GW9662 had no effect on insulin and C-peptide stimulation of PPARγ, confirming its ligand-independent activity (49).

As for PPARα, one study demonstrated that activating human cells with PPARα agonist WY-14 643 led to an upregulation of Th1 cytokines such as IL-1β-induced inflammatory cytokines (50). Furthermore, extracts from PPARα deficient mice demonstrated higher levels of IL13 and GATA-3 (51), which is a vital transcription factor for Th2 differentiation (52). This demonstrates how PPARα promotes the immune reactive state through inhibiting the immune tolerant state of macrophages. However, PPARα has also been shown to be involved in tissue repair. Activation of PPARα using WY 14,643 led to a reduction of acute injury and vascular leakage in perforated mouse lungs (53). Additionally, PPARα activation contributes to rapid repair of intestinal epithelium during SIV infection in macaque models (54). While this does seem contradictory, there is nothing in these studies that suggest that these repairs are related to macrophage polarization, so the idea that PPARα promotes the immune reactive macrophage phenotype is not ruled out.

Out of all the three members of the PPAR family, PPARδ has the least amount of research conducted on it. There is evidence relating PPARδ to the promotion of Th2 cytokines, suggesting its essential relationship with alternative activation of macrophages, however. IL-13 and IL-4 are examples of Th2 cytokines that become active through STAT6 activation (55). Additionally, adipocytes secrete Th2 cytokines involved in alternative activation, as macrophages incubated with adipocyte conditioned medium (CM) displayed an inhibition in pro-inflammatory Th1 cytokines such as MCP-1 and TNFα while displaying an upregulation of immune tolerant marker genes such as Mgl1 and Mgl2 (56). When PPARδ-deficient mice were incubated with adipocyte CM, there was an inhibition of STAT6 activity, inhibiting alternative activation of macrophages. There was also an inhibition of transcription of immune tolerant markers Mgl1, Mgl2, and Mrc2 and an upregulation of Th1 cytokines such as MCP-1, TNFα, and IL-6 (57). This study suggests that PPARδ expression in macrophages is essential for adipocyte-induced activation of immune tolerant state of macrophages. This hypothesis is further corroborated by other studies. GW501516, a PPARδ agonist, inhibits transcription of Th1 cytokines such as IL-6, IL1β, TNFα, and NF-κB as well as neutrophil and macrophage infiltration in mice (58). PPARδ activation has also been shown to suppress IFNγ in mice (58, 59). Another study directly demonstrated that transferring PPARδ-deficient bone marrow into wild type mice led to an inhibition of alternative activation of macrophages (60). We conclude that PPARδ, similarly to PPARγ, promotes the immune tolerant phenotype and inhibits the immune reactive phenotype of macrophages, while PPARα promotes the immune reactive phenotype while inhibiting the immune tolerant phenotype. The members of the PPAR family indirectly regulate each other on their effects on macrophage differentiation through competing cytokines.

## Macrophage Function and PPARs

**Figure 4:**
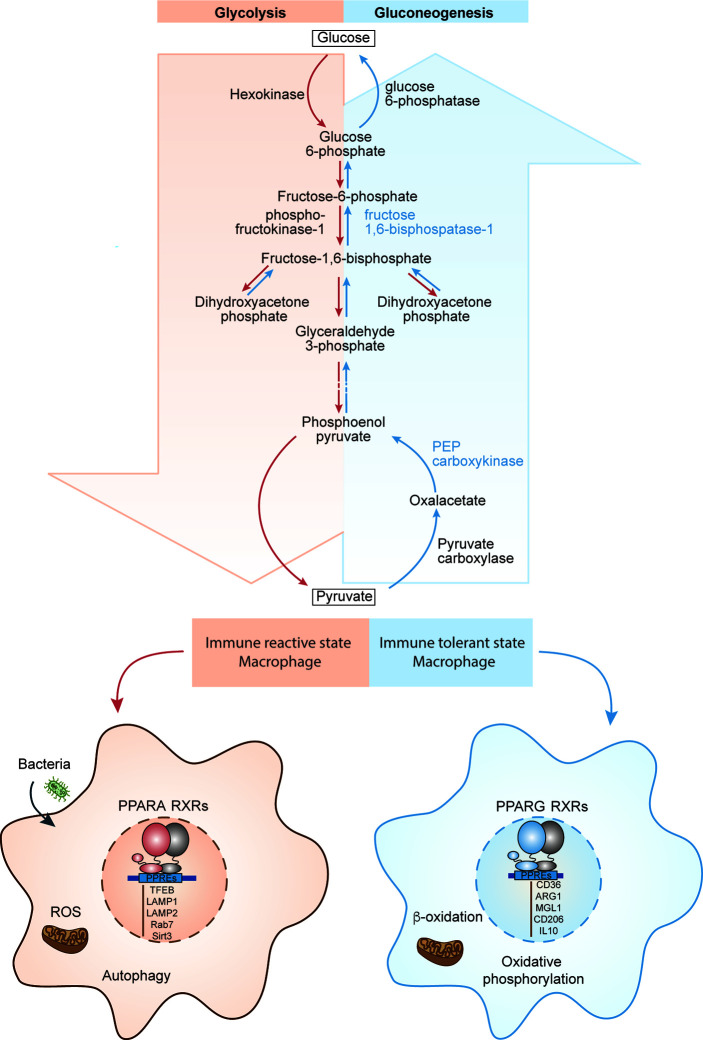
PPARs in macrophage function and polarization. The schematics depicts the biochemical steps involved in glycolysis and glucogenesis, and how each pathway correlates to different immune states of macrophages. PPARA regulates immune reactive state, glycolysis dominant state (red left side) and PPARG regulates immune tolerant state, gluconeogenesis dominant state (blue right side).

Upon infection, macrophages surge at the place of infection and assume a pro-inflammatory, immune reactive state. Immune reactive macrophages are programmed for phagocytosis and killing of the invading pathogen by producing large amount of reactive oxygen species (ROS). Since this infectious environment is low in oxygen, immune reactive macrophages program themselves to survive in low oxygen (hypoxic) conditions (61). Within immune reactive macrophages, both aerobic glycolysis and pentose phosphate pathways are induced upon activation (**Figure 4**). Glycolysis promotes glucose uptake to produce pyruvate (**Figure 4**). However, under hypoxic conditions, NADH cannot be oxidized to NAD+, a required electron acceptor for the further oxidation of pyruvate. Therefore, in hypoxic conditions, pyruvate is first reduced to lactate, accepting electrons from NADH, and thereby regenerating the NAD+ needed for glycolysis to continue (62). In the immune reactive macrophage’s mitochondria, the electron transport chain is dampened, promoting production of mitochondrial reactive oxygen species (mtROS) due to incomplete electron transfers (**Figure 4**). Additionally, induction of pentose phosphate pathways in immune reactive macrophages generates more NADPH, which is needed for the NADPH oxidase to generate cytosolic ROS and nitric oxide (63).

PPARs modulate both glycolysis and NADPH oxidase induced ROS (**Figure 4**). PPARγ diminishes glycolysis in mice by promoting expression of 6-Phosphofructo-2-Kinase/Fructose-2,6-Biphosphatase 3 (PFKFB3), which in gluconeogenesis pathway, converts fructose-2,6-bis phosphate to fructose-6-phosphate and increases accumulation of fructose-6-phosphate (**Figure 4**) (64). This indicates that PPARγ prevents macrophages from polarizing towards the glycolysis dependent immune reactive state, further confirming its anti-inflammatory effect on macrophages. Interestingly, PPARα, but not PPARγ, is essential for NADPH oxidase induced ROS generation in both humans and mice (65). This is further evidence that PPARα contributes to immune reactive macrophage polarization (66).

Immune tolerant macrophages, on the other hand, obtain their energy from fatty acid oxidation and oxidative phosphorylation for tissue repair and tissue remodeling (67). In addition, macrophages can induce the constituent electron transport chain, which is required for oxidative phosphorylation and drives pyruvates into the Krebs cycle (**Figure 4**). Both PPARα and PPARγ promote the gene expression of several molecules/enzymes involved in oxidation of fatty acid. For example, PPARα and PPARγ, in humans, promote expression of mitochondrial fatty acid transporter carnitine palmitoyltransferase 2 (CPT2) and the hydroxyacyl-coenzyme A (CoA) dehydrogenase trifunctional multienzyme complex subunit beta (HADHB) (68), which catalyzes the final step of β-oxidation (**Figure 4**).

Considering PPARα promotes fatty acid oxidation, it may seem as though PPARα is anti-inflammatory. However, the promotion of fatty acid oxidation through PPARα also leads to its inhibition through proinflammatory phospholipid by-products. PPARα has been shown to promote leukotriene B4 (LTB4) synthesis through β-oxidation in rats. Interestingly, LTB4 and other fatty acid derived molecules are ligands for PPARα, thus this feedback loop promotes their own catabolism and leads to resolution of inflammation (69) (**Figure 4**). Even though PPARα contributes to fatty acid oxidation, it only does so to regulate and inhibit itself to allow for less inflammation. Taken together, it emerges again that PPARα is essential for promoting the immune reactive state of macrophage whereas PPARγ is essential for promoting the immune tolerant macrophage. PPARα demonstrates its ability to regulate its own pro-inflammatory abilities through self-inhibition when inflammation resolution is necessary.

## Bacteria and PPARs

When it comes to bacterial infection, PPARγ activation appears to correlate with poor outcome. PPARγ activation in both humans and mice has been shown to decrease the number of neutrophils and macrophages as well as compromise bacterial clearance, which could worsen complications such as influenza-associated pneumonia (70). Several studies have shown that known PPARγ agonists contribute to caspase-associated apoptosis of monocytes, T cells, and B cell progenitors (71–73). PPARγ has also been shown to attenuate neutrophil migration and activation (74). This may explain how PPARγ can cause immunosuppression to the extent of increasing risk of infection. This is further corroborated by a study that demonstrated how PPARγ knockout mice had an increased effector response when infected with *E. coli* (75). This may also explain why PPARγ agonists have shown an increased risk in cardiovascular dysfunction in humans (76), as there are many species of bacteria that can increase risk of cardiovascular disease. PPARγ can certainly be seen as a therapeutic target for infection and immune related diseases but should be done so with the consideration of its indication of poor prognosis through inhibition of bacterial clearance.

On the other hand, PPARα activation has been shown to be protective against bacterial infections. Infection with *Francisella tularensis* heavily upregulates fatty acid metabolism, which we already know is regulated by the PPARα pathway (77). This may be indirect evidence that infection with *F. tularensis* leads to PPARα activation. PPARα activation using gemfibrozil has been shown to decrease the bacterial load of *Mycobacterium tuberculosis* infected mice while also inhibiting *M. abscessus* induced hypersecretion of pro-inflammatory cytokines (78). One more study using mice demonstrated similar results using *Pseudomonas aeruginosa* (79). Another study demonstrated that PPARα-deficient mice have a decreased survival rate during bacterial sepsis as well as impaired liver metabolism (80). Again, in contrast to PPARγ, PPARα demonstrates a pro-inflammatory phenotype, with it promoting cells’ abilities to kill bacteria, especially considering we previously mentioned PPARα’s ability to induce NADPH oxidase formation of ROS, which is essential for bacterial clearance.

As for PPARδ, there is unfortunately a lack of direct evidence on its impact on bacterial clearance. Considering we do know its similarities to PPARγ in its promotion of immune tolerant phenotype macrophages, we can hypothesize that its activation also inhibits bacterial clearance. However, more studies would need to be conducted to confirm this.

## Viruses and PPARs

In infection, too much inflammation can devastate the body. For example, influenza infection can lead to a “cytokine storm”, a hyper-induction of immune response that can lead to complications and lung pathogenesis (81). Considering excessive inflammation is tied to influenza related mortality, PPARγ has been considered as a therapeutic target to limit such harmful inflammation (82). In a recent study, it was demonstrated that in mouse alveolar macrophages, PPARγ mRNA levels were reduced after influenzaA infection and respiratory syncytial virus infection (24). In contrast, the spike protein of SARS-CoV-2 upregulates PPARγ in macrophage-like RAW264.7 cells (83). In another independent study, infection with MERS-CoV upregulated PPARγ in human macrophages (84). It appears that PPARγ is upregulated in certain viral infections while downregulated in other viral infections. Regardless, PPARγ plays a significant role in the prognosis of viral infections in general.

One study demonstrated that PPARγ reduced the secretion of influenza-induced proinflammatory cytokines TNF-a, IL-8, and RANTES in humans (85). PPARγ activation also leads to decreased mortality in obese mice infected with influenza (86). Furthermore, HIV infection in mice also leads to the hyper-induction of proinflammatory genes such as TNFα, IL-1β, IFNγ, CCL2, CCL3, CXCL10, and iNOS, all of which has been shown to be attenuated using PPARγ agonists rosiglitazone and pioglitazone (87). While PPARγ activation leads to poor prognosis in bacterial infection, it apparently leads to good prognosis in viral infection through its inhibition of hyperimmune response.

PPARα activation contributes to rapid repair of intestinal epithelium during SIV infection in macaque models (54). Interestingly, certain viral infections, such as Zika virus, have been shown to modulate and dysregulate PPARα signaling pathways in human cells (88). The core protein of hepatitis C virus (HCV) was also found to inhibit PPARα expression in humans (89). Another study confirmed that both PPARα and PPARγ are downregulated during HCV infection, and then further downregulated during co-infection with HIV in humans (90). Furthermore, activation of PPARα has been shown to inhibit STING activation of type I interferons as well as increase herpesvirus replication in infected mouse cells (91). It appears that PPARα activation and an increase in viral load and pathogenesis are heavily correlated, again showing an inverse relationship between bacterial infection prognosis and viral infection prognosis. PPAR agonists and antagonists may be key therapeutic strategies depending on the type of infection.

## PPARs and Inflammatory Bowel Disease

Inflammatory bowel diseases (IBD) including Crohn’s disease (CD) and ulcerative colitis (UC) negatively impact the quality of life of millions of people (92). CD consists of inflammation of the mouth, anus, and intestines, while UC consists of inflammation in the mucosal layer of the colon (93). Common pro-inflammatory cytokines are associated with IBD such as TNFα, IL-1β, IL-6, IFNγ, and IL-12 (94) which are predominantly secreted by inflammatory immune cells including neutrophils and macrophages. Unsurprisingly, anti-inflammatory drugs are a common treatment for IBD.

Colon RNA seq data revealed the fact that both PPARα and PPARγ are down regulated during IBD disease progression (95, 96). Considering PPARγ demonstrates anti-inflammatory abilities and is highly expressed in the intestines (97), many researchers see PPARγ as a good treatment candidate target. Rosiglitazone, pioglitazone, troglitazone and AS002, known PPARγ agonists, have demonstrated protection and recovery from pathogenic inflammation in colitis mouse models (98, 99) (**Table 1**). However, several PPARγ agonists have failed in clinical trials (**Table 1**).

**Table 1:** PPAR agonists, effects, and market status.

PPAR Agonist	Indications	Effect	Status	Reference
**PPARα Agonist**				
Elafibranor	Atherogenic dyslipidemia, diabetes, obesity	Increases HDL cholesterol, lowers triglycerides and LDL cholesterol, improves insulin sensitivity	Phase III clinical trials	(100)
Lobeglitazone	Diabetes	Reduces blood sugar levels, lowers hemoglobin A1C levels, improves lipid and liver profiles	Approved in South Korea	(101)
WY 14,643	Lipid metabolism, adipogenesis, cell differentiation, inflammation	–	Preclinical	(102)
Pemafibrate	Nonalcoholic fatty liver disease, dyslipidemia	Decreases lipid accumulation	Phase III clinical trials	(103)
Fenofibrate	Primary hypercholesterolemia, mixed dyslipidemia, hypertriglyceridemia	Increases lipolysis and HDL levels, reduces triglyceride levels, cholesterol, and LDL levels	FDA Approved	(104)
Gemfibrozil	Hypertriglyceridemia, dyslipidemia	Increases lipoprotein lipase synthesis and HDL levels, decreases apolipoprotein C-III and LDL levels	FDA Approved	(105)
Bezafibrate	Hyperlipidemia	Decreases LDL levels, increases HDL levels	Phase IV clinical trials	(106)
Omega-3	Hypertriglyceridemia, myocardial infarction	Decreases PGE2 levels and plasma triglyceride levels	FDA Approved	(107)
**PPARγ Agonist**				
Rosiglitazone	Diabetes	Increases insulin-sensitivity, anti-inflammation and NFκβ inhibition Adverse effects: fluid retention, congestive heart disease	Discontinued	(108)
Pioglitazone	Diabetes	Increases insulin sensitivity and blood glucose uptake Adverse effects: congestive heart failure, bladder cancer	Discontinued	(109)
Troglitazone	Diabetes	Antioxidant, vasodilator, anticonvulsant, anticoagulant, and platelet aggregation inhibitor Adverse effects: Liver disease	Discontinued	(110)
AS002	Ulcerative Colitis	–	Preclinical	(98)
AMG-131	Diabetes	Increases insulin sensitivity, decreases blood glucose levels	Phase II clinical trials	(111)
**PPARδ Agonist**				
Seladelpar	Hyperlipidemia, primary biliary cholangitis	Decreases holestatic pruritus and fatigue	Phase III clinical trials	(112)
GW501516	Dyslipidemia, obesity, cardiovascular diseaase	Regulates fatty acid oxidation	Phase II clinical trials	(113)
**PPARα/γ Dual Agonist**				
Muraglitazar	Diabetes	Increases HDL, decreases LDL, triglycerides, and cholesterol Adverse effects: increased risk of heart failure	Discontinued	(114, 115)
Tesaglitazar	Atherogenic dyslipidemia, diabetes	Increase insulin sensitivity Adverse effects: fibrosarcoma	Discontinued	(115, 116)
Naveglitazar	Diabetes	Increases insulin sensitivity	Discontinued	(117)
Ragaglitazar	Diabetes, dyslipidemia	Decreases cholesterol, triglycerides, blood glucose, and LDL, increases HDL	Discontinued	(118)
Farglitazar	Hypoglycemia, hepatic fibrosis	Decreases fibrosis	Discontinued	(119)
Imiglitazar	Diabetes	Decreases hypoglycemic activity Adverse effects: hepatotoxicity	Discontinued	(120)
Netoglitazone	Diabetes	Increases insulin sensitivity	Discontinued	(121)
Reglitazar	Diabetes	Decreases triglyceride levels, protects against neuropathy	Discontinued	(122)
MK0767	Dyslipidemia, diabetes	Increases insulin sensitivity, decreases cholesterol and triglyceride levels	Discontinued	(123)
KRP-297	Diabetes	Reduces lipid oxidation and plasma glucose	Discontinued	(124)
TZD18	Diabetes	–	Preclinical	(125)
Chiglitazar	Dyslipidemia, diabetes	Increases insulin sensitivity	Phase II clinical trials	(126)
Aleglitazar	Diabetes, heart disease	Controls lipid and glucose level with minimal side effects	Phase III clinical trials	(115)
Saroglitazar	Diabetes, non-alcoholic fatty liver disease	Decreases transaminase levels, regulates lipid metabolism, increases insulin sensitivity	Phase II clinical trials, Approved in India and Mexico	(127)
**PPAR Pan Agonist**				
Bavachinin	Metabolic Syndrome	–	Preclinical	(128)
Lanifibranor	Nonalcoholic steatohepatitis	Reduces inflammation, fibrosis, and lipid accumulation	Phase II clincal trials	(129)
MHY2013	Diabetes, hyperlipidemia	–	Preclinical	(130)

Regarding PPARα, there is conflicting evidence on its role in IBD. One study showed how the PPARα-UGT pathway increased *de novo* bile acid synthesis, exacerbating mouse model colitis (131). Another study used a recombinant protein (rSj16) taken from bacteria and demonstrated its effects on inhibiting PPARα as well as protecting against DSS-induced colitis in mice (132). When mouse models were treated with fenofibrate, PPARα activation increased in parallel to colonic inflammation (133). Although, it should be noted that fenofibrates alter many different metabolic pathways (134). One the other hand, several studies conclude the opposite. When mice were treated with PPARα agonist Wy-14643, there was a decrease in susceptibility to colitis (135). Additionally, verbascoside (VB) acts as a collector of intracellular ROS, reducing experimental colitis. PPARα-KO mice showed weaker VB-mediated anti-inflammatory activity compared to wild type, suggesting PPARα’s protective role against IBD (136). Also, in PPARα-KO mice, innate immune cells decreased production of IL-22 and antimicrobial peptides RegIIIβ and RegIIIγ as well as calprotectin. This led to commensal dysbiosis as well as an increased tolerance for gut bacteria that release proinflammatory cytokines (137). Finally, an additional study demonstrated that dexamethasone induced anti-inflammatory activity is weakened in PPARα-KO mice (138).

Knowledge of PPARδ and its role in IBD is severely limited. One study showed that dual activation of PPARδ and PPARγ using conjugated linoleic acid (CLA) downregulated both TNFα and NFκβ activation while upregulating TGF-β1 as well as protecting against DSS and CD4 induced colitis in mice (139). However, another study demonstrated that PPARδ upregulates COX-2 in mouse gut epithelial cells, leading to an increase in macrophage-produced proinflammatory cytokines and increased the risk of colonic inflammation (140).

In our previous study we demonstrated that activation of PPARα or PPARγ individually is not enough for protection against Citrobacter-induced colon infection in mice. However, a dual activation of both PPARα and PPARγ using a balanced dual agonist protected mice form Citrobacter-induced colon infection (141).

Taking all this information, it appears that the use of anti-inflammatory PPARγ agonists prevent excessive inflammation in colon. However, its prolonged use could lead to polarization of gut macrophages towards an immunotolerant state which eventually help the survival and replication of pathogenic gut bacteria and inflate the development of IBDs. Additionally, continuous use of PPARα agonists alone causes excessive activation of NADPH oxidase and mitochondrial dependent ROS production, potentially leading to collateral host tissue damage and inflammation. Therefore, balanced activation of both PPARγ and PPARα is the key to treat the IBD disease and might be help prevent IBD disease progression, combining the pro-inflammatory effects of PPARα and the anti-inflammatory effects of PPARγ.

## PPAR and Atherosclerosis

Atherosclerosis is the leading cause of the development of cardiovascular diseases. During hyperlipidemic conditions, lipids sneak into the subendothelial layer of the aortic wall, where oxidation of lipid alters it to form oxidized LDL (oxLDL). Macrophages scavenge on oxLDL to process it. However, excess oxLDL promotes oxLDL accumulation in macrophages, leading to foam cell formation and atherosclerosis development.

**Figure 5:**
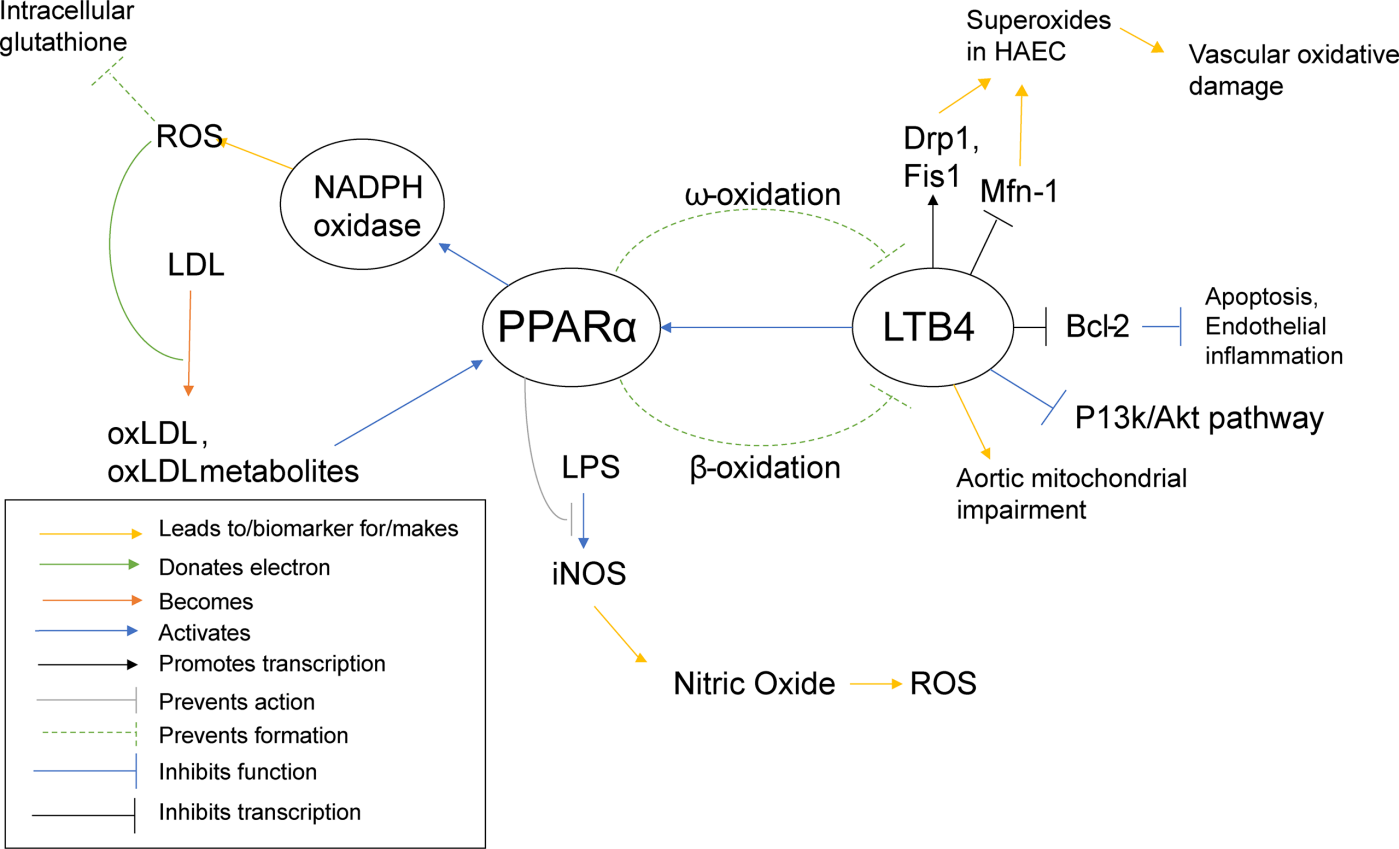
PPARα Inflammatory Pathways. This schematic visualizes how PPARα directly and indirectly influences inflammatory pathways using color coded lines and arrows labeled in the box in the bottom left.

PPARα activation was shown to promote low density lipid (LDL) oxidation in humans and mice (65), as ROS can be responsible for the oxidation of LDL and PPARα increases ROS levels through NADPH oxidase. PPARα activation has also been shown to inhibit LPS activation of iNOS (65), the inducible enzyme that produces nitric oxide (NO). Normally, NO is responsible for overall cardiovascular health (142), vasodilation (143), as well as inhibition of LDL oxidation (144). However, in the presence of an excess of superoxides, NO can react with the superoxides to create peroxynitrite, a reactive peroxide that can lead to ROS and RNS (reactive nitrogen species) (145). Interestingly, oxLDL has been shown to activate PPARα (65). So far, PPARα has been described as pro-inflammatory, yet it also appears to demonstrate self-regulatory abilities. PPARα increases ROS/superoxide levels, as previously stated, and uses oxLDL as a signal to know that such an increase has taken place. In an effort to protect the cell from self-destruction from the creation of too much ROS, PPARα disables an alternate pathway that superoxides can interact with to create even more potentially harmful reactive species, namely the iNOS activation pathway (**Figure 5**).

Polyphenols, such as punicalagin, gallic acid, and ellagic acid, derived from fruits such as pomegranates, have been shown to activate PPARγ activity (146). Activation of PPARγ using these polyphenols increases transcription and protein activity of Paraoxonase 1 (PON1) in HuH7 hepatocyte cells (146). There are also several studies that demonstrate a correlation between an activation of PPARγ, using agonists such as rosiglitazone and several different statins, and an increase in PON1 activity (147–151) (**Table 1**). Once PON1 forms, it associates with high-density lipids (HDL) (152), where is performs several protective functions, such as preventing lipid hydroperoxide formation on HDL as well as protecting the activity of lecithin–cholesterol acyltransferase (LCAT) (153), an enzyme important for HDL antioxidant function and preventing oxidative stress (154). Furthermore, HDL-associated PON1 has been shown to prevent oxidation of low-density lipids (LDL) in humans (155, 156). When LDL is oxidized (oxLDL), lipoperoxides and thiobarbituric acid reactive substances are formed, which can cause oxidative damage. PON1 was shown to inhibit accumulation of these harmful agents in mice (157). By preventing the oxidation of LDL, PON1 and PPARγ demonstrate anti-atherosclerotic properties (158). Furthermore, monocyte chemoattractant protein-1 (MCP-1), which is produced from oxLDL, binds to oxLDL in order to attract macrophages to uptake them. PON1 inhibits this production as seen in human endothelial cells (158). Interestingly, oxLDL also inhibits activity of PON1 in humans (159), possibly in an effort to equilibrate oxidation.

Naturally derived polyphenols have also been shown to increase transcription of paraoxonase 2 (PON2) through activation of PPARγ. This was further confirmed as known PPARγ agonist rosiglitazone was shown to stimulate PON2 expression in mouse macrophages (160). PON2 potentially plays a protective role in the prevention of superoxide and reactive oxygenated species (ROS). Normally within complex 3 of the electric transport chain (ETC), coenzyme Q10 (Q10) donates an electron from QH2 to cytochrome C. Q10’s transition phase, ubisemiquinone, is rather unstable and can sometimes donate the electron to oxygen instead of cytochrome C, when treated with ETC inhibitors, forming superoxides, leading to ROS and oxidative stress (141). Interestingly, PON2 is not only localized within the inner mitochondrial membrane where it is associated with complex 3, but it also binds with high affinity to Q10 (161). Furthermore, PON2 deficient mice were shown to have increased mitochondrial oxidative stress, decreased complex 1 and 3 activities, decreased oxygen consumption, and decreased ATP production (161), demonstrating that lacking PON2 interrupts the ETC. All this information suggests that PON2 associates with Q10, protecting it from destabilization and preventing it from donating electrons to oxygen to form superoxides and ROS. This would mean PON2, and therefore PPARγ, plays an antioxidant role in preventing oxidative stress through the Q cycle pathway.

**Figure 6:**
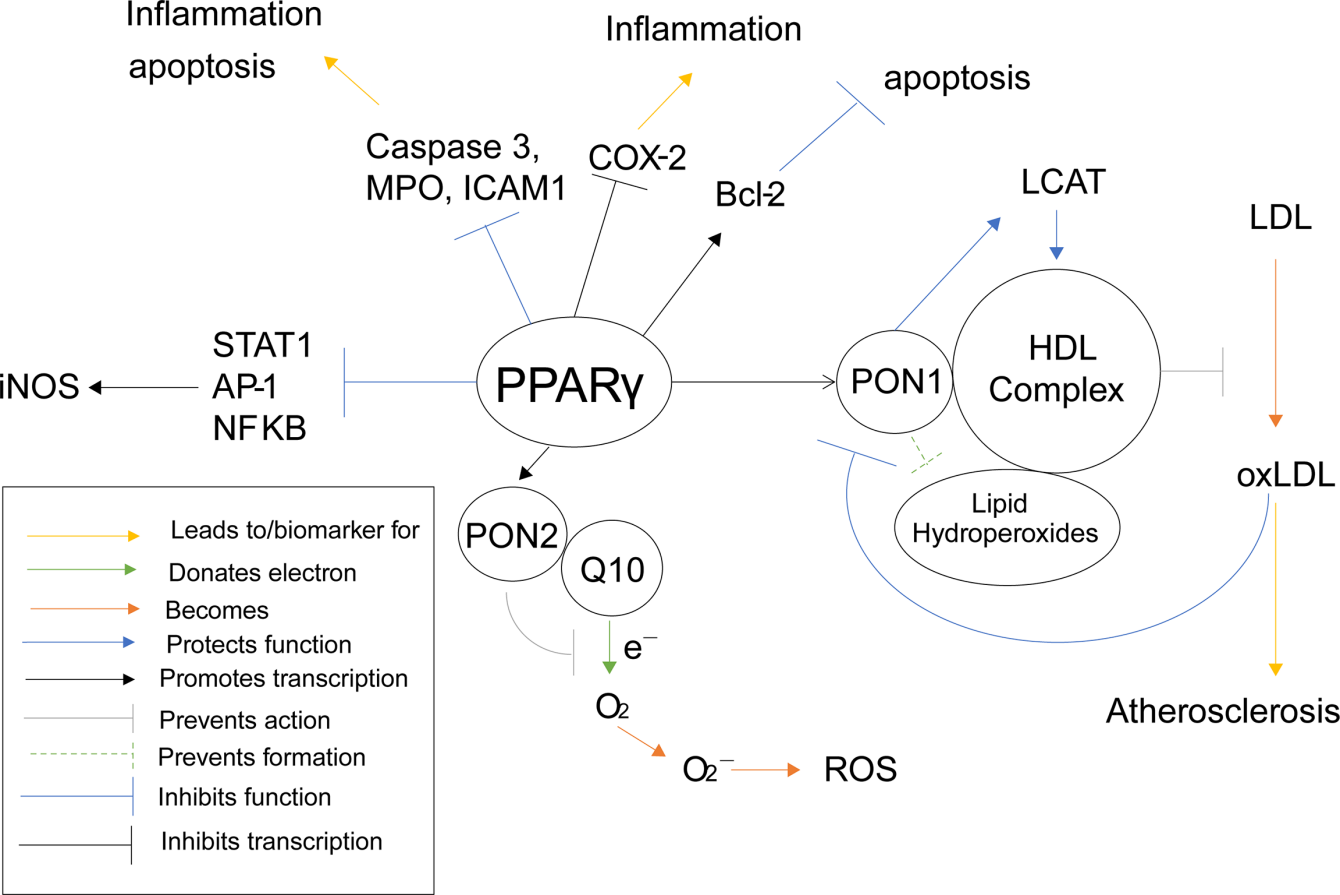
PPARγ Inflammatory Pathways. This schematic visualizes how PPARγ directly and indirectly influences inflammatory pathways using color coded lines and arrows labeled in the box in the bottom left.

PPARγ can also inhibit the production of ROS and oxidative stress in other, more direct ways as well. When mouse macrophages were incubated with PPARγ agonist prostaglandin D2 metabolite 15-deoxy-Δ12,14prostaglandin J2 (15d-PGJ2), the activities of pro-inflammation transcription factors transcription factors AP-1, STAT and NF-κB were antagonized (162). These three proteins act as transcription factors for nitric oxide synthase (iNOS), therefore PPARγ inhibits the transcription of iNOS and the accumulation of nitric oxide (163). Induction of iNOS has been shown to increase ROS levels in mouse RAW264. 7 macrophages as well (164). PPARγ again demonstrates a suppressive role against oxidative stress and ROS (**Figure 6**), which is why it has been such a common target for anti-atherosclerotic therapy. However, considering the previously stated increase in risk of infection, a dual PPARα/γ agonist would be a safer approach, especially considering both PPARγ and PPARα inhibit iNOS related ROS production.

## PPAR Antagonists and Their Therapeutic Potential

PPARγ antagonists such as GW9662 and T0070907 have been shown to inhibition the uptake of apoptotic cells (165). As stated before, immune-suppressive macrophage function includes post-infection repair and cleanup and debris (44). This is further evidence that PPARγ promotes the immune-suppressive phenotype of macrophages. PPARγ antagonist use have also been a strategy for therapy, although less common agonist usage. For example, PPARγ antagonists have shown an ability to increase insulin-sensitivity and as well as inhibit adipocyte differentiation, lipid metabolism, and lipid accumulation in mouse models (166–169). PPARγ antagonist Gleevec has also been shown to increase the browning of white adipose tissue in both humans and mice (170). Because of this, PPARγ antagonists have been seen as a potential therapy for type 2 diabetes and obesity. PPARγ antagonists have also been seen as a potential therapy for cancer, as they have demonstrated antiproliferative effects on cancer cells (171). This is also because fatty acid metabolism is associated with tumorigenesis (172). They have especially been seen as therapy for cancers in areas with high amounts of adipose tissue such as breast cancer (173, 174).

As for PPARα antagonists, there is less research on their therapeutic potential. They have mostly been seen has a potential therapy for different types of cancer. One study demonstrated that PPARα antagonist NXT629 induces apoptosis in chronic lymphocytic leukemia cells using mouse models (175). Another study using mouse models demonstrated that PPARα antagonist GW6471 attenuates tumor growth in renal cell carcinoma (176).

PPARδ antagonists have even less research backing them, but they are generally seen the same way as PPARα antagonists in their use in cancer therapy (177). One study even demonstrates anti-psoriasis therapy in mice (178). Overall, PPAR antagonists are an interesting strategy for therapy, however, there does not seem to be significant clinical research on them (**Table 2**). While there is evidence of their therapeutic benefits, single PPAR antagonists pose the same potential risks of single PPAR agonists. Activating or inhibiting only one member of the PPAR subfamily creates the risk of an overactive or underactive immunity. There is not much research on dual or pan PPAR antagonists, but the use of single PPAR antagonists should be done with these potential risks in mind.

**Table 2:** PPAR antagonists, effects, and market status.

PPAR Antagonist	Indications	Effect	Status	Reference
**PPARα Antagonist**				
TPST-1120	Cancer	Inhibits fatty acid metabolism	Phase I clinical trials	(179)
GW6471	Renal cell carcinoma	Inhibits fatty acid metabolism and glycolysis	Preclinical	(176)
NXT629	Chronic lymphocytic leukemia	–	Preclinical	(175)
MK886	Lung adenocarcinoma	–	Preclinical	(180)
**PPARγ Antagonist**				
GW9662	Cancer, obesity, diabetes	–	Preclinical	(173)
T0070907	Cervical cancer	–	Preclinical	(181)
SR-202	Obesity, diabetes	–	Preclinical	(167)
Betulinic acid	HIV, inflammation, malaria dysplastic nevus syndrome, melanoma	Induces apoptosis, increases ROS and caspase activation	Phase I clinical trials	(182)
Gleevec	Leukemia	Inhibits tyrosine kinase	Approved	(183)
**PPARδ Antagonist**				
GSK-3787	Psoriasis	–	Preclinical	(178)
SR13904	Cancer	–	Preclinical	(177)
GSK0660	Psoriasis	–	Preclinical	(178)

## The Future of PPAR in Therapeutics

In terms of clinical study and treatment, more are leaning towards dual and pan agonists for the PPAR family. While several have been discontinued, several new agonists are in preclinical and clinical trials (**Table 1**). We have demonstrated the dueling relationship between PPARγ and PPARα in terms of macrophage differentiation, bacterial and viral clearance, IBD, and atherosclerosis. A PPARγ/α dual agonist seems to be more promising in terms of therapeutics and activation of both receptors would counter the each other’s side effects while still providing better pharmacological effects (184). Another notable example is how PPARγ agonists have been used as a therapeutic drug for increasing insulin resistance in diabetic patients as well as lipid metabolism in patients with atherosclerosis. However, clinical trials were halted when patients developed increased risks for congenital heart disease (76). While studies of PPARγ/α agonists in relation to diabetes have been done before (185), there has been a greater focus in more recent years on how these dual agonists can treat diabetes with greater efficacy while also limiting the risk of heart failure (76), utilizing both pro and anti-inflammatory effects to our advantage. Another approach for the same problem is using PPARα/δ dual agonists, such as GFT505, which have been shown to treat type 2 diabetes while altogether avoiding the cardiovascular risk of PPARγ agonists (186). The same PPARα/δ dual agonist has also been shown to demonstrate hepatoprotective properties (187). Pan PPAR agonists are being studied for many different conditions such suppressing inflammation and increasing lipid oxidation (188), protecting against metabolic disorders and fibrosis (189), and even angiogenesis in ischemic mice (190).

Regarding direct crosstalk between PPARs, there is unfortunately little evidence. Only one study provided evidence for direct crosstalk, demonstrating that PPARγ inhibits PPARδ while PPARα inhibits PPARδ as PPARδ activates PPARα (191). There is more evidence regarding indirect crosstalk, such as how all three PPARs inhibit NF-κB signaling and function as previously mentioned. There is also how PPARδ increases COX-2 transcription while both PPARγ and PPARα inhibit it. However, looking at indirect relationships gives little insight into direct crosstalk, as there are many interfering pathways. More studies must be done on how specific PPAR activation/inhibition affects other PPAR transcription and activity in order to gain greater insight on the outcomes of PPAR agonists as well as dual and pan agonists.

## Author Contributions

PG and GK conceived and designed the project. DT, PG, and GK drafted, reviewed, and edited the manuscript. All authors contributed to the article and approved the submitted version.

## Funding

This work was supported by the National Institute of Health Grants, AI155696 and AI141630 (to PG). GK was supported through The American Association of Immunologists Intersect Fellowship Program for Computational Scientists and Immunologists.

## Conflict of Interest

The authors declare that the research was conducted in the absence of any commercial or financial relationships that could be construed as a potential conflict of interest.

## Publisher’s Note

All claims expressed in this article are solely those of the authors and do not necessarily represent those of their affiliated organizations, or those of the publisher, the editors and the reviewers. Any product that may be evaluated in this article, or claim that may be made by its manufacturer, is not guaranteed or endorsed by the publisher.
